# Host-Seeking and Sugar-Feeding Behaviors of *Aedes aegypti* in Nouakchott, Mauritania: Implications for Dengue Transmission

**DOI:** 10.3390/tropicalmed11040109

**Published:** 2026-04-21

**Authors:** Mohamed Haidy Massa, Mohamed Aly Ould Lemrabott, Osman Abdillahi Guedi, Nicolas Gomez, Sébastien Briolant, Ali Ould Mohamed Salem Boukhary

**Affiliations:** 1 Unité de Recherche Génomes et Milieux (GEMI), Université de Nouakchott, Nouveau Campus Universitaire, Nouakchott BP 5026, Mauritania; medhaidy@gmail.com (M.H.M.); mohamedalylemrabott@yahoo.fr (M.A.O.L.); alimedsalem@gmail.com (A.O.M.S.B.); 2 Centre de Recherche en Sciences Humaines, Sociales, Langues et Littérature, Université de Djibouti, Campus de Balbala, Croisement RN2-RN5, Djibouti 77101, Djibouti; osman.guedi@gmail.com; 3 Laboratoire de recherches Océan Indien: Espaces et Sociétés (OIES), Université de La Réunion, 97400 Saint-Denis, France; 4 Unité Parasitologie et Entomologie, Département Risques Vectoriels, Institut de Recherche Biomédicale des Armées (IRBA), 13005 Marseille, France; nico13dna@hotmail.com; 5 Unité Mixte de Recherche Risques Infectieux Tropicaux et Microorganismes Emergents, Assistance Publique des Hôpitaux de Marseille, Service de Santé des Armées, Aix Marseille University, 13005 Marseille, France; 6 Institut Hospitalo, Universitaire Méditerranée Infection, 13005 Marseille, France

**Keywords:** *Aedes aegypti*, arbovirus, blood meals, human biting rate, sugar meals, Nouakchott, Mauritania

## Abstract

*Aedes aegypti*, the main urban vector of dengue fever, poses a major public health problem in Nouakchott, Mauritania. This study analyzed the host-seeking and sugar-feeding behaviors of *Ae. aegypti*. Mosquitoes were collected using a vacuum cleaner in four districts between December 2023 and October 2024. Biting activity on humans was studied in May 2024, exclusively in the districts of Ksar, Tevragh Zeina and Arafat, between 5:00 a.m. and 9:00 p.m. A negative binomial model was performed to analyze the effect of location and time on the human biting rate (HBR) of mosquitoes. In Nouakchott, except in the Arafat district, *Ae. aegypti* bites occur mainly outdoors, between 8:00 a.m. and 1:00 p.m., with a peak between 11:00 a.m. and noon (HBR = 20 bites/person), and between 5:00 p.m. and 7:00 p.m., with a peak between 6:00 p.m. and 7:00 p.m. (HBR = 11 bites/person). Inside homes, *Ae. aegypti* biting activity remains low everywhere (HBR ≤ 1.5 bites/person/hour). Molecular analysis of the origin of the blood meals showed that the females collected in Nouakchott were exclusively anthropophilic. Molecular analysis of the sugar sources revealed a great diversity with sweet potato being among the most common. These results highlight the need for targeted outdoor interventions and larval control measures to reduce the risk of dengue transmission in Nouakchott.

## 1. Introduction

Mosquitoes of the genus *Aedes*, particularly *Aedes aegypti* and *Ae. albopictus*, transmit various arboviruses to humans, with dengue posing a persistent global threat. In 2024, worldwide, 14,434,584 cases of dengue, including 7,718,585 laboratory-confirmed cases, 52,738 severe cases and 11,201 deaths, were reported to the WHO [1]. The presence of *Ae. aegypti* in Nouakchott, the capital city of Mauritania, was first reported in 2014 following a routine entomological survey [2]. Shortly thereafter, the species was implicated in a major dengue virus (DENV) epidemic [3]. During the 2014 outbreak in Nouakchott, Fourié et al. confirmed the circulation of serotype DENV-2 [4]. In October 2015, the National Reference Center for Arboviruses (Marseille, France) identified serotype DENV-1 in a French traveler returning from Mauritania [5]. Later, in 2018, DENV-2 was detected by RT-PCR in Ae. aegypti mosquitoes in Rosso, in southwestern Mauritania [6].

The rise of urbanization, globalization, and human migration has led to environmental changes that have contributed to a marked increase in arboviral infections [7]. Arbovirus transmission depends on multiple factors, including mosquito biting behavior, host preference (preferred biting areas, feeding habits, and biting times), as well as the availability and susceptibility of hosts. Mosquito behavior and bite frequency exhibit considerable spatial and temporal variability, with significant differences related to species-specific characteristics and climatic conditions. Therefore, any reliable estimation of biting frequency must account for this heterogeneity [8,9,10]. Furthermore, mosquito activity patterns vary according to the time of day and environmental context, particularly between indoor and outdoor environments [8,9,10,11], as well as between rural and urban areas [12].

*Aedes aegypti* depends on mammalian blood for egg development [13]. This species is primarily anthropophilic and feeds mainly on human hosts during the day [14]. Therefore, understanding its trophic preferences is essential to elucidating its role in disease transmission and to guiding the design of more effective vector control strategies [15,16]. Indeed, studies conducted in countries such as Senegal, Cameroon, and Kenya have shown that *Ae. aegypti* can exploit a wide range of hosts, such as cattle, pigs, rats, dogs, cats, and humans [17,18]. In addition, mosquitoes need sugar to survive, as it provides the energy necessary for flight and other metabolic functions. In all mosquito species, sugar feeding begins shortly after emergence, before the first blood meal, and can continue throughout the gonotrophic cycle [19]. However, despite the considerable burden dengue represents for public health and the recent outbreaks in Mauritania, data on the behavioral ecology of *Ae. aegypti*, particularly its trophic preferences and biting patterns, remain limited.

Therefore, this study aims to describe the host-seeking behavior of *Ae. aegypti* in Nouakchott, in order to obtain blood and sugar.

## 2. Materials and Methods

### 2.1. Study Area and Mosquito Collection Sites

This study was conducted in Nouakchott, the capital of Mauritania. With a population of approximately 1.5 million, the city accounts for nearly one-third of the national population, making it the country’s main urban center [20]. Nouakchott has a hot and arid climate, with temperatures exceeding 27 °C from June to October and averaging 21.4 °C in January. During the study period, temperatures ranged between 21.8 °C and 31.9 °C [21].

Relative humidity varies between 28% and 69% and rainfall is low and irregular (approximately 120 mm annually). The region is characterized by a long dry season (September to June) and a short rainy season (July to September).

Geographically, the city lies on the Atlantic coast in the Saharan zone of Mauritania, a few meters below (−3 m) or above (up to 10 m) sea level. A natural salt belt, one to two kilometers wide, separates the urban area from the Atlantic coast. Seasonal winds shift from northeast (October–May) to northwest (June–September) [22].

**Figure 1:**
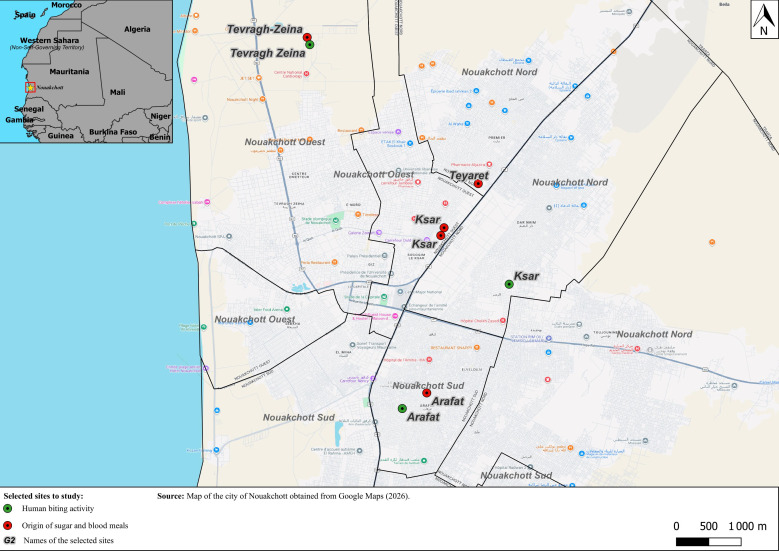
Map showing the sites selected to study Human biting activity (green circles) and to determine the origin of sugar and blood meals (red circles) of *Aedes aegypti* mosquitoes in Nouakchott, the star indicates the location of the city of Nouakchott in Mauritania. Nouakchott Nord, Nouakchott Ouest and Nouakchott Sud correspond to Nouakchott North, Nouakchott West and Nouakchott South, respectively.

Sampling was conducted in four districts—Teyaret, Tevragh Zeina, Ksar, and Arafat (Figure 1 and Table S1)—selected to represent a gradient of urbanization and environmental conditions influencing *Ae. aegypti* ecology. Tevragh Zeina is a highly urbanized and affluent residential area with abundant vegetation (gardens and nurseries), providing potential sugar sources and resting habitats. In contrast, Arafat is densely populated but less urbanized, with limited vegetation. Ksar and Teyaret represent intermediate urban settings. These variations are expected to influence the availability of breeding sites, host exposure, and plant-derived sugar sources for *Ae. aegypti* populations.

### 2.2. Mosquito Collection Methods

#### 2.2.1. Collection of Resting *Aedes aegypti* Using Aspirators

To study feeding habits (sugars and blood), resting adult *Ae. aegypti* mosquitoes were collected between December 2023 and October 2024 using a battery-powered Prokopack aspirator (John W. Hock Co., Gainesville, FL, USA). Sampling was carried out both indoors and outdoors, targeting typical resting habitats of *Ae. aegypti*, including shaded vegetation, walls, ceilings, curtains, clothing, and furniture. These are known resting sites for this species in urban environments. Sampling was carried out systematically using slow upward and downward movements to optimize capture efficiency. Collections were conducted primarily in the morning and late afternoon, times when adult *Ae. aegypti* mosquitoes are more likely to be resting before or after feeding. All collected mosquitoes were transported alive or kept in the laboratory for morphological identification and subsequent molecular analyses.

#### 2.2.2. Human Landing Catch

The host-seeking activity of female *Ae. aegypti* mosquitoes was assessed using the human landing capture (HLC) method at Tevragh Zeina, Ksar, and Arafat. Sampling was conducted over six consecutive days in May 2024, in the absence of a dengue epidemic, with two days per site, from 5:00 a.m. to 9:00 p.m., thus covering periods of high daytime activity of *Ae. aegypti*. This method targets female mosquitoes seeking a host to feed on blood. Mosquitoes landing on exposed body parts were captured before they bite using hand-operated mouth aspirators. The captured mosquitoes were transferred to labeled plastic containers covered with netting, indicating the date, time, location, and whether the collection took place indoors or outdoors. The samples were then transported to the laboratory for identification and further analysis. Each collection session involved four trained volunteers (two indoors and two outdoors). To minimize bias related to physical appearance, the volunteers regularly alternated positions (for one hour indoors for two volunteers and one hour outdoors for the other two; then they switched places, and so on throughout the session; during the next session, they proceeded in the same way but reversed their positions compared to the first session). They wore protective clothing that only exposed their forearms and legs, the areas most frequently bitten by *Ae. aegypti* mosquitoes.

### 2.3. Mosquito Identification

In the laboratory, mosquitoes were morphologically identified as *Ae. aegypti* using a stereomicroscope and standard dichotomous identification keys [23]. Identification was based on characteristic features such as the lyre-shaped white markings on the thorax and banded legs.

Only *Ae. aegypti* specimens were retained for subsequent analyses of host-seeking behavior, blood meals, and sugar-feeding patterns.

### 2.4. Ethical Considerations

The experimental protocol for this study was reviewed and formally approved by the research ethics committee of the University of Nouakchott (No. 2023-0050). Informed consent was obtained from the volunteers before their enrolment.

### 2.5. Molecular Identification of the Origin of Sugar and Blood Meals

Seventy-three blood-engorged female *Ae. aegypti* mosquito specimens were used for blood meal analysis and 737 *Ae. aegypti* specimens for sugar meal analysis. Plant and mosquito DNA was extracted from mosquito abdomens. The abdomens were placed in microtubes containing 5 mm diameter stainless steel beads and 205 μL of lysis buffer (25 μL of proteinase K and 180 μL of T1 buffer supplied in the NucleoSpin^®^ 96 Tissue Core Kit) for use with the TissueLyser II system (Qiagen S.A.S., Courtaboeuf, France). The samples were homogenized in the TissueLyser II for three one-minute cycles at a frequency of 30 Hz, with 20 s pauses between each cycle. The samples were then incubated at 70 °C for one hour. DNA extraction was performed using the NucleoSpin^®^ 96 Tissue Core Kit (Macherey-Nagel AG, Oensingen, Switzerland) according to the manufacturer’s instructions. Two PCR reactions were performed. The first PCR amplified plant DNA by targeting the ribulose-1,5 bisphosphate carboxylase (rbcl) gene. A fragment of approximately 402 base pairs was amplified using the following primers. Forward: TCGTCGCAGCG-TCAGATGTGTATAAGAGACAGTGGCAGCATTYCGAGTAACTC. Reverse: GTCTCGTGGGCTCGGAGATGTGTAT-AAGAGACAGCYAAYARGGGACGACCA. The second PCR amplified mitochondrial DNA by targeting the cytochrome oxidase I (COI) gene. A fragment of approximately 663 base pairs was amplified using primers Forward: AACCACAAAGACATTGGCAC and Reverse: AAGAATCAGATARGTGTTG.

Three microliters of eluted template DNA was added to the PCR master mix containing DreamTaq DNA polymerase, reaction buffer, 2 mM MgCl2, and dNTPs (ThermoFisher DreamTaq™ Green PCR Master Mix; ThermoFisher Scientific, Illkirch, France). The thermocycler program (T1 Biometra, Thermo Fisher Scientific, Illkirch, France) was as follows: an initial denaturation at 95 °C for 5 min, followed by 35 cycles of 95 °C for 30 s, 55 °C (annealing temperature) for 30 s, and 72 °C for 1 min, followed by extension at 72 °C for 7 min. Purification of PCR products and sequencing were outsourced to Microsynth SAS (Vaulx-en-Velin, France). Finally, all sequences were compared to NCBI databases by BLAST to identify the plant sources of sugary meals or the origin of blood meals.

### 2.6. Statistical Analysis

Data were entered and saved in Excel, then analyzed with R software version 4.4.2 [24]. A generalized linear mixed model (GLMM) with the negative binomial family (lme4 package) was performed to study the effect of location (outdoors/indoors) and capture time (with a random effect on operator and site) on the biting rate of *Ae. aegypti* female mosquitoes. The biting rate of female *Aedes* mosquitoes was expressed as the average number of bites per person per hour. The number of people corresponded to the number of participants used as human bait.

## 3. Results

### 3.1. Origin of Blood Meals

Analysis of blood meals revealed that the 73 female mosquitoes collected in the three districts of Nouakchott (Teyarett, Arafat, and Ksar) had fed exclusively on human blood.

### 3.2. Origin of the Sugar Meals

The analyses were carried out on 737 mosquitoes collected in four districts of Nouakchott: Ksar, Tevragh-Zeina, Teyaret, and Arafat. The number of specimens analyzed by PCR varied according to the site (Table 1). A single band corresponds to the appearance of a single DNA fragment after migration on an agarose gel, indicating that the mosquito likely fed on a single plant. Conversely, the presence of several DNA fragments of varying sizes, or multiple bands, suggests that the mosquito fed on several plants.

The Ksar site provided the largest number of mosquitoes analyzed (587). The single-band PCR success rate was 11.2%. Seven samples were successfully sequenced, representing 1.2% of the initial sample. From Tevragh-Zeina, 101 specimens were analyzed, 27 individuals gave single-band PCR results (26.7%) and 11 were successfully sequenced (approximately 11%). Thirty-nine mosquitoes were analyzed in Teyaret, 3 gave positive single-band PCRs, of which two could be sequenced (5.1%). At the Arafat site, 10 mosquitoes were analyzed, six produced a positive single-band PCR, but none could be sequenced.

From the 20 sugar-fed mosquitoes with successful *rbcl* gene sequences (Table 1), analysis of the plants on which mosquitoes had fed revealed a wide variety of plant families, particularly in Tevragh-Zeina and Ksar (Table 2). Among the most common plants, the sweet potato (*Ipomoea batatas*, *Convolvulaceae* family) stands out in Tevragh-Zeina, where six mosquitoes fed on it, making it the most attractive plant in this sample.

The same botanical family was also represented by *Porana velutina*, which attracted three mosquitoes in both Tevragh-Zeina and Ksar. The African oil palm (*Elaeis guineensis*, *Arecaceae* family) in Ksar attracted two mosquitoes, as did the banana tree (*Musa acuminata*, *Musaceae* family) in Teyaret. Several other plants attracted only one mosquito at a time, including Chinese weeping cypress (*Callitropsis funebris*) at Tevragh-Zeina, false mimosa (*Leucaena leucocephala*) and peanut (*Arachis hypogaea*) at Tevragh-Zeina, corn (*Zea mays*), also at Tevragh-Zeina, African foxtail (*Ceratotheca triloba*), stone pine (*Pinus pinea*) and *Lithocarpus longzhouicus* in Ksar.

In terms of spatial distribution, Tevragh-Zeina has the greatest diversity of plants that serve as a sugar source for mosquitoes, with seven different species. Ksar follows with five species, although the number of sugar-fed mosquitoes is lower for most plants there. Teyaret has only one identified plant (the banana tree) and only two mosquitoes were observed there.

### 3.3. Biting Activity Cycle

No effect of the operator was observed on the biting rate of female *Ae. aegypti* mosquitoes (GLMM with a negative binomial distribution, *p* = 0.38). The biting rate of female *Ae. aegypti* mosquitoes outdoors was significantly higher than indoors (GLMM with a negative binomial distribution, *p* = 0.03).

**Figure 2:**
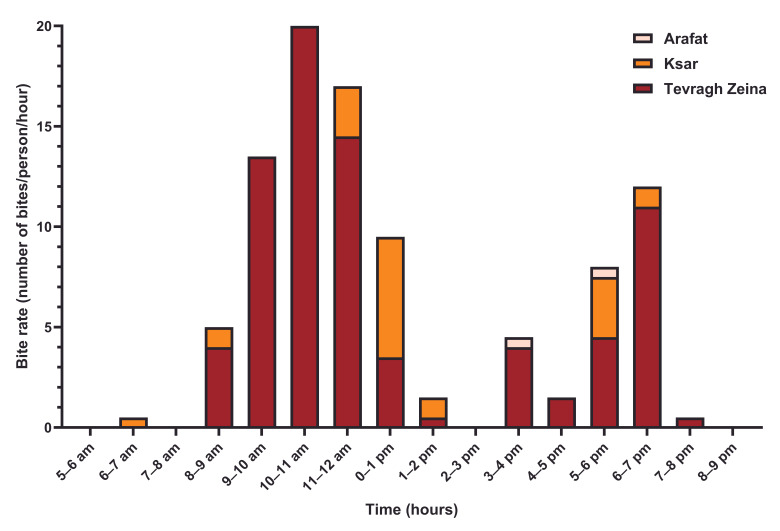
Outdoor biting activity cycle of *Aedes aegypti* female mosquitos in three district of Nouakchott.

**Figure 3:**
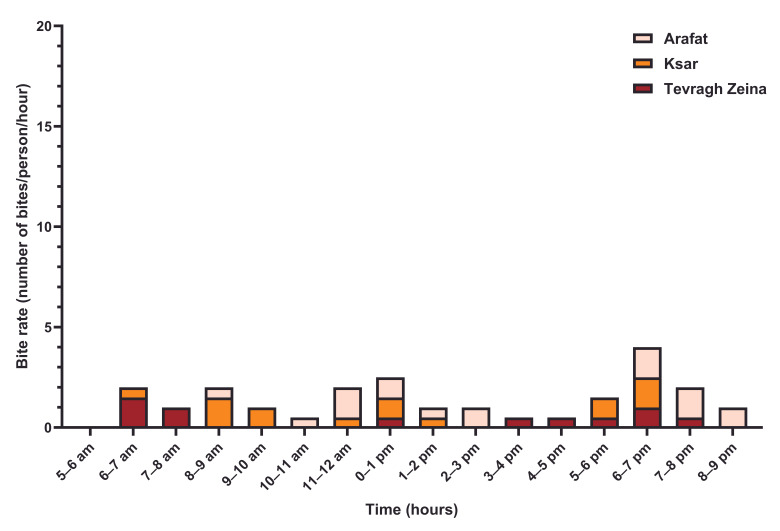
Indoor biting activity cycle of *Aedes aegypti* female mosquitos in three districts in Nouakchott.

In Tevragh Zeina, two periods of activity of female *Ae. aegypti* mosquitoes were observed outside (Figure 2), between 8:00 a.m. and 2:00 p.m. with a peak between 10:00 a.m. and 11:00 a.m. (20 bites/person), and between 3:00 p.m. and 8:00 p.m. with a peak between 6:00 p.m. and 7:00 p.m. (11 bites/person). Inside homes, mosquito activity remained low throughout the day, with two identical peaks in intensity (1.5 bites per person), between 6:00 a.m. and 7:00 a.m. and between 6:00 p.m. and 7:00 p.m. (Figure 3). In the Ksar district, the highest rates of outdoors bites (Figure 2) were observed between noon and 1:00 p.m. (6 bites/person) and between 5:00 p.m. and 6:00 p.m. (3 bites/person). Inside homes, *Ae. aegypti* biting activity remained low, not exceeding 1.5 bites/person/hour, with three periods between 6:00 a.m. and 10:00 a.m., between 11:00 a.m. and 2:00 p.m. and between 5:00 p.m. and 7:00 p.m. (Figure 3). In the Arafat district, female *Ae. aegypti* mosquitoes hardly bite outdoors except between 3:00 p.m. and 4:00 p.m., and between 5:00 p.m. and 6:00 p.m. (Figure 2). Biting activity remained low indoors, not exceeding 1.5 bites/person/hour, with two periods between 10:00 a.m. and 3:00 p.m., and between 6:00 p.m. and 9:00 p.m. (Figure 3).

## 4. Discussion

In this study, the *Ae. aegypti* populations of the three districts studied exhibited a clear bimodal biting pattern, characterized by a marked peak in the late morning, shortly before noon, and a second peak at sunset. This diurnal rhythm is consistent with the results of previous laboratory and field studies. For example, experimental work conducted in Trinidad revealed two peaks of activity, one between 6:00 a.m. and 10:00 a.m. and the other between 3:00 p.m. and 6:00 p.m. [25]. Similarly, Chompoosri et al. observed comparable peaks in the morning and afternoon in Thailand [26].

However, regional variations have been observed. In northern Ghana, a late morning peak has been reported without a clearly defined afternoon peak [8], suggesting that biting rhythms may be influenced by ecological and climatic factors. Our results also indicate that biting activity primarily occurs outdoors, confirming the exophagic tendency of *Ae. aegypti* described in studies conducted in Ethiopia [27], Ghana [8], and Trinidad [12]. A comparable behavior has been observed in the *Ae. simpsoni* complex in Ethiopia [28]. In contrast, Casas-Martínez et al. [9] observed higher biting activity inside buildings, highlighting the plasticity of this species and its ability to adapt to local environmental conditions.

The observed biting behavior, particularly outdoors, can be influenced by human activities and the presence of potential attractants, such as light or food sources, during peak hours. The low frequency of bites inside homes is explained not only by implemented vector control measures but also by the limited access mosquitoes have to buildings. Furthermore, residents spend a significant portion of their time outdoors, especially in May, a warm period that increases the risk of mosquito contact. These observations suggest that the interaction between mosquito behavior and human habits must be considered to better understand the risk of transmission.

The exclusive detection of human blood meals in female *Ae. aegypti* mosquitoes in Nouakchott confirms a marked anthropophilic behavior. This result is consistent with numerous studies reporting extremely high proportions of human blood meals in urban environments. In Thailand, this proportion has reached 99 to 100% in some urban settings [29]. Similar observations have been reported for *Ae. albopictus*, with 100% human blood meals in some localities in southern Thailand. In Senegal, 78.6% of the 1710 blood meals analyzed were of human origin despite the presence of domestic animals, and anthropophily can reach 90% depending on the season and location [30]. In Ouagadougou (Burkina Faso), more than 90% of blood meals came from humans in several health districts [31]. Together, these results highlight the strong adaptation of *Ae. aegypti* to human hosts in urban African and Asian contexts.

In this study, most *Ae. aegypti* mosquitoes have likely not fed recently. This could explain why only 18.5% (136/737) of the PCR tests were positive. In fact, most researchers tested for the presence of fructose—an indicator of recent feeding on plants—using the cold anthrone test, before performing PCR to amplify and sequence the *rbcl* gene. They generally obtained fructose positivity rates ranging from 2.8% for *Anopheles* [32] to 44.7% for sandflies [33], depending on whether the insects were captured indoors or outdoors, whether they were females or males, etc. In one study, the fructose positivity rate was 21% for female *Ae aegypti* [34], which is not far from our results. Regarding sugar feeding, our results demonstrate that *Ae. aegypti* exploits a wide variety of urban plant species in Nouakchott, particularly in Tevragh-Zeina, Ksar, and Teyaret. Among the plants identified are *Ipomoea batatas*, *Porana velutina*, *Elaeis guineensis*, and *Musa acuminata*, highlighting the opportunistic nature of this species in its choice of sugar sources. *Ipomoea batatas* proved particularly attractive, with six sugar-laden specimens recorded in Tevragh-Zeina. These observations are consistent with studies from Mali and Kenya demonstrating that *Ae. aegypti* readily feeds on nectar-bearing plants in urban environments [35,36]. The wide diversity of plant species identified suggests that control strategies targeting a single attractive species may prove insufficient. Furthermore, integrating repellent or less attractive plant species into urban landscaping could constitute a complementary approach to vector management.

Several limitations should be taken into account. The sampling was conducted over a relatively short period and was limited to three neighborhoods, which may not fully reflect the seasonal or spatial variability in mosquito behavior in Nouakchott. The number of engorged females analyzed was small, and the low positivity rate of sugar feeding (*rbcl* PCR positivity as a proxy) as well as the low success rate of plant identification through sequencing may have led to an underestimation of floral diversity. One limitation of this study is that direct sequencing primarily identifies the dominant food source without reflecting the full diversity of the diet; the use of next-generation sequencing (NGS) could improve this characterization. Another limitation of this study is that the lack of a qualitative and quantitative assessment of available plant resources limits the ability to determine whether the identified plants reflect true dietary preferences or simply environmental availability. Furthermore, dengue virus infection rates have not been assessed. Larger, longitudinal, city-wide studies are therefore needed to validate and expand upon these findings.

### Implications for Dengue Transmission

The behavioral characteristics of *Ae. aegypti* observed in Nouakchott have important implications for the transmission dynamics of dengue. The strongly anthropophilic diet of *Ae. aegypti* indicates intense contact between humans and the vector, which could promote sustained viral circulation in urban environments. The bimodal biting activity, with a major peak in the late morning and a second peak at dusk, coincides with periods of increased human activity, thus increasing the risk of exposure. The predominance of outdoor bites in some districts suggests that indoor interventions, such as spraying with long-lasting insecticides, may have limited effectiveness if implemented alone. Furthermore, the opportunistic exploitation of various sugar sources could improve mosquito survival and longevity, potentially increasing their vector capacity. The development of repellent creams could be a complementary strategy for reducing contact between mosquitoes and humans. However, since the mosquitoes studied feed on various attractive plants as a source of sugars, in this context, developing plant-based products that are not attractive to mosquitoes remains a challenge. Further investigations are needed to assess the transmission dynamics of dengue in Nouakchott: larval density indices in the studied areas as well as possible correlation between the number of dengue cases in the last few years in the study areas and the densities and biting behaviour of *Aedes* vectors.

## 5. Conclusions

This study is the first to describe some bionomic aspects of *Ae. aegypti* in the urban area of Nouakchott, Mauritania, in particular its aggressive and trophic behavior, including both blood and carbohydrate feeding. The *Ae. aegypti* mosquito exhibits bimodal biting activity, with a significant peak in the late morning, just before noon, and a second peak at sunset. *Aedes aegypti* is primarily exophagic. This anthropophilic behavior, which involves feeding strongly on humans, increases the likelihood of interactions between humans and the vector and, consequently, the risk of disease transmission, especially since the biting periods closely coincide with the daily routines of the population of Nouakchott, while *Ae. aegypti* also adopts opportunistic behavior to feed on sugar. These results underscore the need for integrated dengue control strategies in Nouakchott. These approaches should combine targeted outdoor interventions, reduction in community-level sources of contamination, and, potentially, the use of attractive toxic sugar baits (ATSB), in addition to conventional larval control measures, to effectively reduce the risk of transmission.

## Figures and Tables

**Table 1:** Number of *Aedes aegypti* mosquito captured and results of molecular analyses for *rbcl* gene.

Site	Number of Mosquitoes Analyzed	Single-Band PCR	Multi-Band PCR	Number of Samples Successfully Sequenced
Ksar	587	66	26	7
Tevragh-Zeina	101	27	6	11
Teyaret	39	3	1	2
Arafat	10	6	1	0

**Table 2:** Sugar-feeding plants of *Aedes aegypti* in four study district in Nouakchott, Mauritania.

Family	Species	Common Name	Mosquito Capture Site	Number of Mosquitoes Feeding on the Plant
*Arecaceae*	*Elaei guineensis*	African oil palm	Ksar	2
*Convolvulaceae*	*Ipomoea batatas*	Sweet potato	Tevragh-Zeina	6
*Convolvulaceae*	*Porana velutina*	ND	Tevragh-Zeina, Ksar	3
*Cupressaceae*	*Callitropsis funebris*	Chinese weeping cypress	Tevragh-Zeina	1
*Fabaceae*	*Leucaena leucocephala*	Leucaena	Tevragh-Zeina	1
*Fabaceae*	*Arachis hypogaea*	Peanut	Tevragh-Zeina	1
*Fagaceae*	*Lithocarpus longzhouicus*	ND	Ksar	1
*Musaceae*	*Musa acuminata*	Banana	Teyaret	2
*Pedaliaceae*	*Ceratotheca triloba*	African foxglove	Ksar	1
*Pinaceae*	*Pinus pinea*	Pin parasol	Ksar	1
*Poaceae*	*Zea mays*	Maize	Tevragh-Zeina	1

ND: not determined.

## Data Availability

The data presented in this study are available on request from the corresponding author.

## Supplementary Materials

The following supporting information can be downloaded at: https://www.mdpi.com/article/10.3390/tropicalmed11040109/s1, Table S1: Geographical location of the sites studied in the four wilayas of Nouakchott.

## Abbreviations

The following abbreviations are used in this manuscript:

DENV	Dengue virus
DNA	Desoxyribonucleic acid
GLMM	Generalized linear mixed model
PCR	Polymerase chain reaction
