# Supplementary material for: Host-Seeking and Sugar-Feeding Behaviors of Aedes aegypti in Nouakchott, Mauritania: Implications for Dengue Transmission
Source: Trop Med Infect Dis. 2026 Apr 21;11(4):109. doi: 10.3390/tropicalmed11040109 (PMC13120532; doi:10.3390/tropicalmed11040109)
Supplement: Supplementary file 1 [file tropicalmed-11-00109-s001.zip › Table S1.pdf]

**Table S1.** Geographical location of the sites studied in the four wilayas of Nouakchott.

| Wilaya | Moughataa | Type of study | Latitude | Longitude |
|---|---|---|---|---|
| SN | Arafat | Human biting activity | 18.049987 N | 15.965711 W |
| WN | Ksar | Human biting activity | 18.086919 N | 15.933954 W |
| WN | Tevragh-Zeina | Human biting activity | 18.160250 N | 15.993972 W |
| SN | Arafat | Blood and sugar meals | 18.054631 N | 15.958440 W |
| SN | Arafat | Blood and sugar meals | 18.049987 N | 18.049987 N |
| WN | Ksar | Blood and sugar meals | 18.103429 N | 15.952836 W |
| WN | Ksar | Blood and sugar meals | 18.103696 N | 15.953298 W |
| WN | Tevragh-Zeina | Blood and sugar meals | 18.160250 N | 15.993972 W |
| NN | Teyaret | Blood and sugar meals | 18.116801 N | 15.943138 W |

NN: North Nouakchott; WN: West Nouakchott; SN: South Nouakchott.
